# Effect of Daratumumab combined with Chemotherapy on Immune Function in Patients with Relapsed/Refractory Multiple Myeloma and Observation of its Clinical Efficacy

**DOI:** 10.12669/pjms.39.1.6667

**Published:** 2023

**Authors:** Ye-hua Zhang, Feng Xu, Chang-qing Xu, Zi-tan Zhang, Zong-jiu Jiao

**Affiliations:** 1 Ye-hua Zhang, Department of Hematology, Xingtai People’s Hospital, Xingtai 054001, Hebei, China; 2 Feng Xu, Department of Hematology, Xingtai People’s Hospital, Xingtai 054001, Hebei, China; 3 Chang-qing Xu, Department of Hematology, Xingtai Third Hospital, Xingtai 054000, Hebei, China; 4 Zi-tan Zhang, Department of Hematology, Xingtai People’s Hospital, Xingtai 054001, Hebei, China; 5 Zong-jiu Jiao, Department of Hematology, Xingtai People’s Hospital, Xingtai 054001, Hebei, China

**Keywords:** Daratumumab, Relapsed/Refractory multiple myeloma, Immune function, Treatment

## Abstract

**Objective:**

To evaluate the clinical efficacy and immune function of Daratumumab combined with chemotherapy in patients with relapsed/refractory multiple myeloma (RRMM).

**Methods:**

Eighty patients with RRMM treated in Xingtai People’s Hospital from January, 2020 to December, 2021 were randomly divided into two groups. Patients in the study group were treated with Daratumumab combined with PAD regimen, while patients in the control group were provided with PAD regimen alone. Further comparison was performed on the therapeutic effects, adverse drug reactions, the levels of T lymphocyte subsets CD3^+^, CD4^+^, CD8^+^ and CD4^+^/CD8^+^, the positive expression rate of CD38 and the expression level of Notch-1 on the membrane of plasma cells between the two groups.

**Results:**

The overall response rate in the study group (67.50%) was significantly better than that in the control group (45.00%). There was no significant difference in the incidence of adverse reactions between the two groups. After treatment, the reviewed levels of CD3^+^, CD4^+^ and CD4^+^/CD8^+^ were obviously higher in the study group than those in the control group, while the positive expression rate of CD38 and the expression level of Notch one on the membrane of plasma cells were both lower than those in the control group (p<0.05).

**Conclusion:**

Daratumumab combined with a PAD regimen is a safe and effective approach that has a definite curative effect on patients with RRMM, which can improve immune function significantly and result in no significant increase in adverse reactions.

## INTRODUCTION

Multiple myeloma (MM) is a B-cell malignancy featured by the clonal proliferation of malignant plasma cells, accounting for 10% of all hematological malignancies.^1^ There is a high incidence of MM in populations aged between 63~70 years old.^2^ It is characterized typically by abnormal plasma cells secretion and monoclonal immunoglobulin production, leading to bone marrow (BM) hematopoietic failure, and finally anemia, osteolytic lesions, hypercalcemia, hyperviscosity syndrome, renal failure, recurrent bacterial infection, etc.^3^ MM is still incurable at present, with a high risk of recurrent attacks. Patients may gradually form resistance to chemotherapeutics, immunomodulators and proteasome inhibitors, and finally relapsed/refractory MM (RRMM).^4^ RRMM has been a clinical challenge. Due to the complex and diverse pathogenesis of this tumor, multi-target therapy has been gradually applied clinically, such as new immunomodulators, proteasome inhibitors, monoclonal antibodies, histone deacetylase inhibitors, inhibitors of nuclear export, etc.^5^

In recent research, CD38 has been found to be highly expressed in MM, highlighting the great potential of anti-CD38 monoclonal antibodies for the treatment of MM.^6^ Daratumumab has broad-spectrum killing activity. It induces anti-MM effect via binding to transmembrane extracellular enzyme CD38 on the membrane of MM plasma cells to induce MM cell lysis.^7^ In our practice, patients with RRMM were treated by Daratumumab combined with chemotherapy, with the achievement of certain clinical efficacy.

## METHODS

A total of 80 patients with RRMM treated in Xingtai People’s Hospital from January, 2020 to December, 2021 were included and randomly divided into two groups, with 40 cases in each group. There was no significant difference in general data between groups, indicating the existence of comparability between the two groups (Table-I). The study was approved by the Institutional Ethics Committee of Xingtai People’s Hospital on March 15, 2020 (No.[2020]039), and written informed consent was obtained from all participants.

**Table-I:** Comparative analysis of general data between the study group and the controls group (*χ̅*±*S*) n=40.

Indexes	Study group	Control group	t/χ^2^	p
Age (years)	65.50±3.84	65.70±4.47	0.215	0.831
Male (%)	23 (57.50%)	25 (62.50%)	0.208	0.648
***Number of lesions***			1.039	0.595
2 sites	16 (40.00%)	15 (37.50%)		
3 sites	13 (32.50%)	17 (42.50%)		
>3 sites	11 (27.50%)	8 (20.00%)		
***Clinical stage (DS stage)***			0.978	0.613
Stage I	17 (42.50%)	19 (47.50%)		
Stage II	14 (35.00%)	10 (25.00%)		
Stage III	9 (22.50%)	11 (27.50%)		

P>0.05.

### Inclusion criteria:

- Patients with RRMM, i.e., those who had relapsed and need repeated treatment after achieving the curative effect of minimal response (MR) or above, or those who had progressed disease within 60 days from the last treatment^8^;
- Patients aged ≥18 years and <70 years old;
- Patients with complete clinical data;
- Patients with normal physical condition and independent living ability (KPS score ≥80 points);
- Patients with an expected survival of over six months;
- Patients who could cooperate to complete the study and had good treatment compliance;
- Patients without contraindications to the drugs used in this study.

### Exclusion criteria:

- Patients with other blood system diseases;
- Patients with malignant tumors of other organs;
- Patients with abnormal liver and kidney function;
- Patients who took drugs that might affect the research results in the near future, such as hormones and immunosuppressants;
- Pregnant or lactating patients;
- Patients with primary refractory MM.

The patients in the study group were treated with Daratumumab combined with chemotherapy. The specific methods were described as follows: Intravenous drip of 16 mg/kg Daratumumab, once a week in the first three weeks (one day, eight day and 15 days); administration on the first day of the 4th~9th week, once every three weeks; once every four weeks after the 9th week. One course of treatment lasted for eight weeks. PAD regimen was used for chemotherapy, with intravenous injection of 1.3 mg/m^2^/d Bortezomib on the 1st, 4th, 8th and 11th day of treatment; intravenous injection of dexamethasone (20 mg per day) on the 1^st^~4th and 8^th^~11th days of treatment; and intravenous injection of 15 mg/m^2^/d Doxorubicin on the 1^st^~4th days of treatment. One course of treatment lasted for 11 days, and patients were given a continuous treatment of 2~8 courses, with an interval of three weeks between the two courses.

The patients in the control group were provided with a PAD regimen alone. During the treatment, the patients in both groups were checked for routine blood tests, liver and kidney function examinations, ECG and chest radiographs. During the treatment, antibiotics were given according to the specific situation of the patients. After treatment, patients in both groups were followed up for half a year to compare the clinical efficacy.

### Clinical efficacy included:

***Complete remission (CR):*** After treatment, patients were detected with negative results of serum M protein test (lasted for > 6 weeks), the disappearance of plasmacytoma, the content of bone marrow plasma cells of < 5%, and no aggravation in the osteolytic lesion;

***Near-complete remission (nCR):*** After treatment, patients were detected with a positive result of immunofixation electrophoresis, the disappearance of plasmacytoma, the content of bone marrow plasma cells of < 5%, and no aggravation in the osteolytic lesion;

***Partial remission (PR):*** After treatment, the patients were detected with the content of serum M protein decreased by >50% (lasted for > six weeks), quantification of the 24 h urinary light chain quantity of <200 g or the extent of decrease of >90% (lasted for > six weeks), the reduction of plasma cell tumor of >50%, and no aggravation in the osteolytic lesion. Meanwhile, the patients with non-secretory MM were detected with the content of plasma cells decreased by >50% by bone marrow puncture or biopsy (lasted for > six weeks); Total overall response rate = (CR+nCR+PR)/the number of total cases ×100%.^9^

The adverse drug reactions of the two groups after one treatment cycle were recorded, including fever, rash, bone marrow suppression, gastrointestinal reaction, liver function injury, fatigue, etc.

Fasting blood was taken in the morning before and after treatment to detect the levels of T lymphocyte subsets CD3^+^, CD4^+^, CD8^+^ and CD4^+^/CD8^+^, so as to compare and analyze the differences between the two groups.

Positive expression rate of CD38 and the expression level of Notch one on the membrane of plasma cells: Before treatment and after one course of treatment, a bone marrow puncture was performed to collect 2ml of bone marrow fluid. The rate of CD38+ plasma cells and the expression level of Notch one on the membrane of CD38 plasma cells were detected by surface antigen labeling using flow cytometry.

### Statistical analysis:

All data were analyzed by SPSS 20.0 software, and the measurement data are expressed in (*χ̄*±*S*). Independent sample t-tests were used for inter-group data analysis, paired t-test for intra-group data analysis, and χ^2^ tests for rate comparison. *P*<0.05 meant the presence of a statistically significant difference.

## RESULTS

The comparative analysis results of clinical efficacy between the two groups is shown in Table-II. The overall response rate in the study group (67.50%) was significantly better than that in the control group (45.00%), and the difference was statistically significant (p=0.043). As shown in Table-III, according to the comparative analysis of the incidence of adverse drug reactions between the two groups after treatment, there was no statistical difference between groups (p=0.478).

**Table-II:** Comparative analysis of clinical efficacy between the two groups (*χ̅*±*S*)n=40.

Groups	CR	nCR	PR	MR	NC	PD	Overall response rate
Study group	8	8	11	7	4	2	27 (67.50%)
Control group	6	9	3	9	7	6	18 (45.00%)
χ^2^							4.114
p							0.043

*P<0.05.

**Table-III:** Comparative analysis of the adverse drug reactions between the two groups after treatment (*χ̅*±*S*) n=40

Groups	Fever	Fatigue	Bone marrow suppression	Rash	Gastrointestinal reaction	Liver function injury	Incidence
Study group	3	2	2	3	3	2	15 (37.50%)
Control group	2	2	3	1	2	2	12 (30.00%)
χ^2^							0.503
p							0.478

p>0.05.

After treatment, the reviewed levels of CD3^+^, CD4^+^ and CD4^+^/CD8^+^ were obviously higher in the study group than those in the control group, and the difference was statistically significant (p=0.000). While there were no obvious changes of CD8+ in the two groups before and after treatment (p>0.05).Table-IV.

**Table-IV:** Comparative analysis of T lymphocyte subsets between the two groups before and after treatment (*χ̅*±*S*)n=40.

Indexes		Study group	Control group	t	p
CD3+ (%)	Before treatment	42.66±6.45	42.86±6.19	0.149	0.882
	After treatment*	48.57±6.37	43.29±6.14	3.776	0.000
CD4+ (%)	Before treatment	27.31±4.12	27.52±3.83	0.236	0.814
	After treatment*	38.26±4.66	34.07±4.84	3.944	0.000
CD8+ (%)	Before treatment	21.46±3.71	21.72±3.72	0.313	0.755
	After treatment*	22.05±3.73	22.18±3.88	0.153	0.879
CD4+/CD8+	Before treatment	1.28±0.10	1.28±0.07	0.225	0.823
	After treatment*	1.76±0.18	1.55±0.16	5.255	0.000

* p <0.05.

After treatment, the positive expression rate of CD38 and the expression level of Notch one on the membrane of plasma cells were both reduced in both groups than those before treatment, which were lower in the study group than those in the control group, with statistically significant difference (p<0.05).Table-V.

**Table-V:** Comparative analysis of the positive expression rate of CD38 and the expression level of Notch1 on the membrane of plasma cells in the two groups before and after treatment (*χ̅*±*S*)n=40.

Indexes	Time	Study group	Control group	t/χ^2^	p
Positive expression rate of CD38 (%)	Before treatment	40 (100.00%)	40 (100.00%)	0.000	1.000
	After treatment*	9 (22.50%)	20 (50.00%)	6.545	0.011
Notch1 expression in plasma cells (n×10^-2^)	Before treatment	58.71±7.44	58.63±6.90	0.050	0.960
	After treatment*	31.49±7.95	42.55±7.26	6.497	0.000

* p<0.05

## DISCUSSION

The pathogenesis of MM is quite complex, which may be affected by genetic factors, environmental factors, virus infection, etc.,^10^ leading to excessive production of M protein and malignant proliferation of bone marrow plasma cells. These patients may have dual defects of humoral immunity and cellular immunity that can escape the immune killing function of the body. Therefore, despite a continuous improvement in the remission rate of MM due to the application of new chemotherapeutics, immune agents, targeted drugs and other therapeutic choices, MM patients still have a five-year survival rate of 44% after initial diagnosis, and it will eventually relapse and develop into RRMM. At present, there are still no satisfactory therapeutic drugs for patients with RRMM.^11^ RRMM is difficult to treat, with a high drug resistance rate and low remission rate., and the current research priority is to find a new treatment scheme.^12^

It has been confirmed that the PAD regimen is effective in the treatment of MM.^13^ Previous research^14^ has reported that Bortezomib can coordinate the interaction between bone marrow tumor cells and stromal cells, which, however, cannot provide benefit for patients with various adverse cytogenetic abnormalities. Furthermore, glucocorticoid is the basic pharmacotherapy in the treatment of MM.^15^ It can have a synergistic effect with other drugs and reverse the drug resistance of MM to chemotherapy drugs chemotherapeutics.

Despite the emergence of multiple new therapeutic drugs in recent decades, RRMM still has a poor prognosis. It was discovered that CD38 was highly expressed in mm, and anti-CD38 monoclonal antibody offers a promising alternative to the treatment of MM.^16^ Daratumumab can directly bind to CD38 on the membrane of myeloma cells and induce myeloma cell death through multiple mechanisms, including antibody-dependent cell-mediated cytotoxicity, complement-dependent cytotoxicity, antibody-dependent cell phagocytosis and direct apoptosis, thereby achieving rapid remission of the disease.^17^ CD38-targeted Daratumumab represents a major milestone in the development of RRMM immunotherapy.

Furthermore, multiple clinical trials have shown that Daratumumab has reliable clinical efficacy and good safety in the treatment of RRMM.^18^ In 33% of RRMM, the application of Daratumumab combined with dexamethasone can improve the induction effect, which can enhance the clinical benefits and improve the overall survival rate compared with monotherapy.^19^ In addition, Offidani^20^ reported that the use of Daratumumab-involved quadruple therapy for treating RRMM exhibited obvious clinical effects, without a significant increase in the incidence of adverse reactions.

Our study confirmed that the study group using the quadruple therapy of Daratumumab combined with PAD regimen had an overall response rate of 67.5%, and that of the control group was 45%, with a statistically significant difference (p=0.04); it also showed no obvious increase in the rate of adverse reactions (p=0.48); obviously higher levels of CD3^+^, CD4^+^ and CD4^+^/CD8^+^ than those in the control group after treatment (p=0.00); and lower positive expression rate of CD38 and the expression level of Notch one on the membrane of plasma cells than those in the control group (p=0.00).

### Limitations

It includes the relatively smaller sample size and no follow-up. With the deepening of research, our further research may be performed based on large sample size and the collection of follow-up data, so as to evaluate the long-term effect and benefits of the proposed therapeutic approach objectively.

## CONCLUSIONS

Daratumumab combined with a PAD regimen is a safe and effective approach that has a definite curative effect for the treatment of patients with RRMM, showing obvious improvement of immune function and no significant increase in adverse reactions.

### Authors’ Contributions:

**YZ** **and** **ZJ** designed this study, prepared this manuscript, are responsible and accountable for the accuracy and integrity of the work.

**FX and** **CX** collected and analyzed clinical data.

**ZZ** Data analysis**,** significantly revised this manuscript.

## References

[1] Shaikh SP, Irfan SM, Sheikh SS (2019). Disease staging according to international scoring system in newly diagnosed patients with multiple myeloma. Pak J Med Sci.

[2] Kazandjian D (2016). Multiple myeloma epidemiology and survival:A unique malignancy. Semin Oncol.

[3] Bridoux F, Leung N, Belmouaz M, Royal V, Ronco P, Nasr SH (2021). Management of acute kidney injury in symptomatic multiple myeloma. Kidney Int.

[4] Chim CS, Kumar SK, Orlowski RZ, Cook G, Richardson PG, Gertz MA (2019). Management of relapsed and refractory multiple myeloma:novel agents, antibodies, immunotherapies and beyond [published correction appears in Leukemia.

[5] Castillo JJ (2019). Evolution of Therapy for Relapsed/Refractory Multiple Myeloma. J Natl Compr Canc Netw.

[6] Saltarella I, Desantis V, Melaccio A, Solimando AG, Lamanuzzi A, Ria R (2020). Mechanisms of Resistance to Anti-CD38 Daratumumab in Multiple Myeloma. Cells.

[7] Davies F, Rifkin R, Costello C, Morgan G, Usmani S, Abonour R (2021). Real-world comparative effectiveness of triplets containing bortezomib (B), carfilzomib (C), daratumumab (D), or ixazomib (I) in relapsed/refractory multiple myeloma (RRMM) in the US. Ann Hematol.

[8] Chari A, Richardson PG, Romanus D, Dimopoulos MA, Sonneveld P, Terpos E (2020). Real-world outcomes and factors impacting treatment choice in relapsed and/or refractory multiple myeloma (RRMM):a comparison of VRd, KRd, and IRd. Expert Rev Hematol.

[9] Riccomi G, Fornaciari G, Giuffra V (2019). Multiple myeloma in paleopathology:A critical review. Int J Paleopathol.

[10] Ali MU, Maqsood S, Malik M, Bano K (2021). Central nervous system involvement in Multiple Myeloma-Diagnosis treatment and outcome:A case report. J Pak Med Assoc.

[11] Cho SF, Anderson KC, Tai YT (2018). Targeting B Cell Maturation Antigen (BCMA) in Multiple Myeloma:Potential Uses of BCMA-Based Immunotherapy. Front Immunol.

[12] Dimopoulos MA, Lonial S, White D, Moreau P, Weisel K, San-Miguel J (2020). Elotuzumab, lenalidomide, and dexamethasone in RRMM:final overall survival results from the phase 3 randomized ELOQUENT-2 study. Blood Cancer J.

[13] Roussel M, Moreau P, Hebraud B, Laribi K, Jaccard A, Dib M (2020). Bortezomib, thalidomide, and dexamethasone with or without daratumumab for transplantation-eligible patients with newly diagnosed multiple myeloma (CASSIOPEIA):health-related quality of life outcomes of a randomised, open-label, phase 3 trial. Lancet Haematol.

[14] Sonneveld P, Avet-Loiseau H, Lonial S, Usmani S, Siegel D, Anderson KC (2016). Treatment of multiple myeloma with high-risk cytogenetics:a consensus of the International Myeloma Working Group. Blood.

[15] Burwick N, Sharma S (2019). Glucocorticoids in multiple myeloma:past, present, and future. Ann Hematol.

[16] Zannetti BA, Faini AC, Massari E, Geuna M, Maffini E, Poletti G (2020). Novel Insights in Anti-CD38 Therapy Based on CD38-Receptor Expression and Function:The Multiple Myeloma Model. Cells.

[17] Cavo M, San-Miguel J, Usmani SZ, Weisel K, Dimopoulos MA, Avet-Loiseau H (2022). Prognostic value of minimal residual disease negativity in myeloma:combined analysis of POLLUX, CASTOR, ALCYONE, and MAIA. Blood.

[18] Chehab S, Panjic EH, Gleason C, Lonial S, Nooka AK (2018). Daratumumab and its use in the treatment of relapsed and/or refractory multiple myeloma. Future Oncol.

[19] Kaufman JL, Dimopoulos MA, White D, Benboubker L, Cook G, Leiba M (2020). Daratumumab, lenalidomide, and dexamethasone in relapsed/refractory myeloma:a cytogenetic subgroup analysis of POLLUX. Blood Cancer J.

[20] Offidani M, Corvatta L, Morè S, Nappi D, Martinelli G, Olivieri A (2021). Daratumumab for the Management of Newly Diagnosed and Relapsed/Refractory Multiple Myeloma:Current and Emerging Treatments. Front Oncol.

